# Exploring the determinants of Suicide among Married Women in Chitral, Khyber-Pakhtunkhwa, Pakistan

**DOI:** 10.12669/pjms.41.5.9319

**Published:** 2025-05

**Authors:** Jafaryad Hussain, Sahil Sajjad, Kausar Hussain

**Affiliations:** 1 Ms. Jafaryad Hussain, MSN, MPH. Department of Nursing, City University Peshawar, Pakistan; 2 Mr. Sahil Sajjad, BSN, MPH. Department of Nursing, City University Peshawar, Pakistan; 3 Ms. Kausar Hussain, M.Phil. Department of Education, University of Chitral, Pakistan

**Keywords:** Domestic violence, Factors of suicide, Family ethnography, Self-destructive behaviour

## Abstract

**Objectives:**

This study aimed to explore the factors that lead to committing suicide among married women of Chitral, Khyber-Pakhtunkhwa.

**Method:**

A qualitative study using a family ethnographic approach was utilized to explore the complex factors contributing to suicidal behavior among women in Chitral, aiming to understand the underlying causes and risk factors. Data collection took place between April to July 2016, employing a combination of semi-structured interviews, observation, and study records. To analyze the gathered data, a social-ecological model was used, which led to the identification of various categories and themes. It is important to note that the study received approval from the ethical committee of Aga Khan University Karachi, ensuring compliance with ethical guidelines. To protect the privacy and confidentiality of the participants, pseudonyms were assigned, ensuring anonymity throughout the research process.

**Results:**

According to the research findings, domestic violence was the primary reason for suicide among the women, which was further divided into five categories, including: (i) personal factors; (ii) family level factors; (iii) community level factors; (iv) society level factors;

**Conclusion:**

Study findings provided insights into determinants leading to suicide among married women in Chitral. The major contributing factor highlighted is domestic violence with sub-factors. The findings of the research study will make significant contributions to the prevention of suicide among women.

## INTRODUCTION

Suicide is a deliberate action that results in the death of the individual and is a significant contributor to global mortality, causing almost one million deaths annually.^1^ Suicide has a devastating global impact on youth, claiming the lives of many young people. It is the second leading cause of death worldwide among 15–29-year-olds and the third leading cause among the economically productive age group of 15–44-year-olds. In Australia, the situation is equally concerning, with over 2,000 suicides occurring every year, making it the leading cause of death for individuals under 44 years old. 2

The global suicide rate is 14.5 per 100,000 population. National and international reports confirm that about 150/100000 women lose their lives due to suicide in India and Pakistan annually.^3^ According to World Health Organization (WHO) estimates, Pakistan reported 13,377 suicides in 2012, translating to a suicide rate of 7.5 per 100,000 population per year. Additionally, the WHO suggests that for every reported suicide, there are approximately 20 attempted suicides. This implies that the actual number of attempted suicides in Pakistan may be significantly higher, ranging from 130,000 to 270,000 annually.^4^

A study in India found a significant link between domestic violence (DV) and attempted suicide in married women of reproductive age. DV increases the risk of attempted suicide by nearly four times. Other risk factors include young age, poor social support, family history of psychiatric disorders, and husband’s alcohol use disorder. Islamic faith was found to be protective.^5^ A study on suicidal tendencies among women found a significant link with experiences of violence and substantial variations in suicidality rates across different sites, including 15-fold variations in suicide attempts and 4-7-fold variations in suicidal thoughts.^6^

Case-control studies and psychological autopsies of suicide cases have investigated and identified mental illnesses, low socio-economic status, loneliness, life events, disrupted social networks, level of academic qualification, marital status, and depression as risk factors for suicide in Pakistan.^7^ Another study indicates that women exposed to intimate partner violence (IPV) are more likely to develop depression, while those already struggling with depression are at increased risk of experiencing IPV. Furthermore, the study underscores an alarming link between IPV and suicidal behavior, highlighting the critical need for targeted interventions to break this cycle of violence and trauma.^8^ Research underscores the critical link between domestic violence victimization and suicidal behavior. A striking correlation emerged between domestic violence and suicide attempts among women. Those with a history of suicide attempts exhibited significantly higher levels of domestic violence exposure compared to those without such attempts.^9^

A disturbing trend has emerged in Chitral, where a growing number of women are taking their own lives. According to a local media report, there is an alarming trend in suicide rates in Chitral, with 176 reported cases between 2013 and 2019. Notably, the research found that young people and women are disproportionately affected, with 144 victims aged 15-30 and 58% being young women, indicating a disturbing shift in suicide demographics.^10^ Despite the escalating number of female suicides in Chitral, the government and relevant authorities have failed to take decisive action to address this critical issue. No concerted efforts have been made to investigate the underlying causes of these tragedies or implement preventive measures. The media’s coverage has been superficial, merely reporting the escalating numbers without probing the complex factors driving these incidents. The lack of adequate mental health facilities in the region has created a vacuum, leaving vulnerable individuals without access to essential support services. Furthermore, the health department’s inadequate reporting mechanisms have contributed to the underreporting of these cases. The stigma associated with mental health issues in the community has also led to a culture of silence, preventing affected families from seeking help or disclosing the factual circumstances surrounding these tragic events.^10^

This study seeks to conduct an in-depth examination of the complex and interconnected factors that contribute to suicidal behavior among married women residing in Chitral, a district located in the Khyber-Pakhtunkhwa province of Pakistan. The primary objective is to identify and understand the underlying causes, motivations, and risk factors that drive married women in this region to contemplate and attempt suicide

## METHODS

A qualitative study design was employed, utilizing a family ethnographic approach to explore the causes of suicide among women in Chitral. This approach focused on the perceptions and experiences of immediate family members of women who had died by suicide in the past year. Data was collected through non-numerical methods, including In-depth interviews with family members to gather detailed accounts of their experiences and perspectives, observations of family dynamics and cultural practices to provide context and insight, and checking of reports from police and human rights departments. The study took place in the district of Chitral, Khyber Pakhtunkhwa, Pakistan. Ethnography is a research method that immerses the researcher within the subject’s culture to gain an in-depth understanding.^11^,^12^ Twenty-four immediate family members of six dead women were the population for this study.

### Ethical Considerations:

### Ethics Review Committee (ERC) Approval:

This study was conducted after obtaining approval from the Ethics Review Committee (ERC) of Khan University Karachi, letter number Ref. No: 4030-SON-ERC-16, Dated: May 11, 2016.

### Informed Consent:

Participants were informed of the study’s purpose, risks, benefits, and confidentiality before participating. The consent form was translated into the local language.

### Participant Anonymity:

To preserve participant anonymity, the following measures were taken:

- Participants were assigned a unique identifier code and kept their personal information confidential.
- Data was stored securely, using password-protected electronic files and locked physical storage.

### Inclusion and exclusion criteria:

The selection criteria were developed through a multi-step process, involving a comprehensive literature review, expert consultations, stakeholder engagement, data analysis, and criteria development, to select study participants and ensure a thorough understanding of suicidal behavior among women in Chitral.

- Family members of ladies who were present with women within one week of women’s suicide.
- *Age:* Women aged 15-45 years.
- *Residence:* Women residing in Chitral district.
- *Timeframe:* Suicides occurring within one year.

During the study period, a total of 12 women succumbed to suicide. However, data saturation was achieved after conducting in-depth interviews with 24 immediate family members of the suicide cases, providing a rich and comprehensive understanding of the complex factors surrounding these tragic events.

### Sampling Technique:

A purposive sampling technique was utilized to recruit the immediate family members of women who had committed suicide.

### Identification of Cases:

Potential participants were identified through the following sources:

- Medical records of women who presented to the hospital with suicidal deaths were reviewed.
- Local community health workers, traditional birth attendants, and other stakeholders were engaged to identify women who had died due to suicide.
- Human resource and police departments were approached to trace reported cases of female suicide in Chitral during the previous year.

### Data Collection Technique:

To maintain the highest standards of research rigor and transparency, this study employed a multi-faceted approach, combining in-depth interviews, observational data, and official records from the police department. To ensure the integrity and effectiveness of the data collection process, the researcher underwent comprehensive training in qualitative research methods, cultural sensitivity, ethics, and confidentiality. Furthermore, a pilot test of the questionnaire was conducted before the data collection, allowing for refinement and optimization of the research instrument.

### In-depth Interviews:

The in-depth interviews with the 24 immediate family members were conducted by a single researcher, who is a trained and experienced qualitative researcher. This approach ensured consistency and continuity throughout the data collection process. Fortunately, all approached participants willingly shared their experiences, and no refusals were encountered. Data saturation was achieved after conducting in-depth interviews with 24 immediate family members, indicating that no new themes or insights emerged, and the collected data was sufficient for meaningful analysis.

The researcher conducted in-depth interviews with participants in the comfort of their homes to gather detailed, contextual information about their experiences. This approach allowed for a personalized and non-threatening environment, encouraging participants to share their thoughts and experiences openly. Each interview lasted approximately 45-60 minutes. The duration of the interviews allowed for an in-depth exploration of the participants’ experiences, thoughts, and feelings regarding the cases.^11^-^14^

### Observation:

Observations were conducted weekly, for 2-3 hours per session, over six months. During these observations, meticulous records were made of the participants’ non-verbal cues, including body language, facial expressions, working environment, behavior of family members, household activities, and family responsibilities. These observations encompassed posture, gestures, eye contact, tone of voice, and physical interactions, providing valuable insights into the participants’ emotional states and experiences. The observations were systematically recorded in field notes and later used to triangulate the data, increase validity, and identify patterns and themes. This approach ultimately provided a richer understanding of the research phenomenon, offering a more comprehensive understanding of the participants’ lives**.**

### Data from the police station and Human Resource Department:

The researcher gathered secondary data from the police department and human resource department through archival research and official records requests. Following obtaining necessary approvals from the relevant authorities, the researcher accessed and analyzed records of suicide cases within the district, providing valuable contextual information for the study

### Assessment Tools:

A comprehensive interview protocol was developed, translated into the local language, and pilot-tested to ensure systematic and rigorous data collection. A semi-structured interview guide was designed, informed by existing literature, to cover relevant topics while allowing for flexibility and probing. Additionally, a sociodemographic questionnaire was administered to gather information on participants’ background characteristics, providing a comprehensive understanding of their socioeconomic context and informing the interpretation of findings.

### Data Analysis:

The study employed a rigorous and systematic approach to data analysis, involving *verbatim* transcription of interviews, careful examination and coding of transcripts to identify concepts, themes, and ideas, and grouping of codes based on shared ideas and concepts through a process known as “constant comparison.” This iterative process continued until no new information emerged and codes and categories reached saturation. To further validate findings, additional data was collected through document review and participant observation, allowing the researcher to gather more information, triangulate findings, and gain a deeper understanding of participants’ experiences and behaviors, ultimately providing a rich and comprehensive understanding of the research phenomenon.^4^,^15^

## RESULTS

The information collected was transcribed exactly as it was recorded. The data was then organized into themes and categories. It is important to clarify that in this context, the term ’case’ refers to the woman who died by suicide, and the term ’participant’ refers to the family members of the woman. The demographic data indicates that their mean age was 27 years. Half of the cases (50%) were illiterate. Another striking commonality among these women was their occupation - all were housewives, dedicating their time to domestic responsibilities. Additionally, they all lived in a joint family system, residing with extended family members, with household sizes ranging from 5 to 11 members. Their spouses were employed as farmers, with a monthly income ranging from Rs. 5000 to Rs. 10000.

### Domestic Violence:

The social-ecological model was employed to analyze the factors contributing to violence. It identified four levels: individual, family/relationship, community, and society. This model acknowledges the complex interactions between these levels and factors in shaping violent behavior.^16^

### Individual Level Factors:

Infertility and depression became the contributing factors to violent behavior towards women, leading to cases of suicide.

*‘The fear of not being able to conceive a child caused distress, with husbands resorting to physical abuse and threats of divorce if she failed to give birth within a one-year timeframe.” ((Brother))*

I am worried that I will never have a child and that my husband will marry again because he has told me that if I do not conceive during this one year, he will go for another marriage. My mother-in-law also threatens me with bringing another daughter-in-law. I am trying to get treatment from here and there, but all are useless. While crying she said that now she has no choice, so she will have to die to get rid of this hell-like life. (Sister)

Depression and lack of grooming contributed to the violence towards ladies that resulted in ending their lives. While sharing her views about the case one of the participants stated:

She was kind to other people but she was not doing anything for herself. She was kept crying, sleeping, or sitting alone. She did not like to go to social gatherings instead she would sit at home alone. She was not taking care of her health, her diet, and her hygiene. They would call her (Mukul) monkey. (Sister)

### Family Level Factor:

Family/relationship factors such as lack of parental support, mismatched marriages, and extramarital affairs played a role in contributing to suicide among women. While expressing feelings about the case, one participant shared the exact *verbatim*:

*“I am so unlucky that my mum is no longer able to listen to my problems. My husband beats me every day but I cannot do anything. I do not have any way to get rid of this difficult life except killing myself” (Sister In-law)*

While sharing his experience of lack of father’s support for the case, a participant stated:

Whenever my kai (sister) came to our home, she met us with an upset mind and cried while leaving. My father is not telling the truth. He is a dictator; therefore, my sister was uncomfortable talking about her family issues while sharing with us (younger brothers and sisters). When I tried to tell my father about the situation, he stopped me by saying that it was her own choice to marry so she was responsible for her difficult life. He further verbalized that I could not do anything by bypassing my father, thus I kept quiet and my sister committed suicide. (Brother)

Regarding the extramarital affair of the case’s husband, the participant stated:

She discovered that her husband was involved with another woman, which she found unacceptable. When she confronted him about his unfair behavior and demanded fair treatment, it became a challenge to his sense of masculinity. In response, he subjected her to severe physical punishment, using a bat and breaking several rough sticks while beating her.” (Brother

### Community Level Factors:

The lack of community support was identified as a significant factor contributing to women’s suicides. Women experiencing physical and psychological pain had no one to listen to their voices and provide support.

### A participant shared about her sister’s situation before suicide:

Despite her deteriorating health, her in-laws refused to let her visit a hospital. Additionally, her husband, who had no interest in having children, subjected her to daily abuse. She sought support from her relatives and parents, but her pleas went unnoticed. Feeling trapped with no other options, she tragically took her own life.” (Sister)

### Another participant discussed the lack of support from community-level organizations, sharing the following account:

She was exhausted from her husband’s daily abuse. Despite sharing her ordeal with her parents, they advised her to seek justice from the local council. However, after approaching the council, no action was taken. Unfortunately, this led to an escalation in her husband’s frequency of violence towards her. (Sister-in-law)

Restrictions on women’s social actions, such as being barred from visiting parents and friends, praying, or using the phone, contributed to suicidal tendencies.

Second wife of the husband of a case) shared her experiences:

I am alone in my home, and my husband is away the entire day. He doesn’t allow me to go outside without his permission. However, when he returns, he ignores me and shows no respect. I have no one to talk to at home, and I’m not allowed to visit my parents or relatives. I’m unable to share my feelings with anyone. I’m constantly thinking about my bad luck and crying. Whenever I ask him for permission to visit my parents for a few days or meet my relatives, he warns me that if I leave, I should be prepared to never come back. (Second wife) In a household with over 10 family members, my daughter faced immense challenges in caring for everyone and completing all tasks single-handedly. She requested her husband for a separate home to alleviate the burden, but he refused and resorted to violence. Overwhelmed and exhausted, she tragically took her own life. (Mother)

### Society-Level Factors:

Research findings have highlighted that societal factors, such as high expectations placed on women and adherence to traditional gender roles, contribute significantly to violent behavior and subsequent suicides among women in Chitral.

Participants shared their experiences regarding gender roles and societal expectations of women;

In a household with over 10 family members, my daughter faced immense challenges in caring for everyone and completing all tasks single-handedly. She requested her husband for a separate home to alleviate the burden, but he refused and resorted to violence. Overwhelmed and exhausted, she tragically took her own life. (Mother)

A participant while defending his brother and father for beating the case stated:

I think they (my brother and father) did not do anything wrong (that they hit her) because running the home properly, cooking, washing, cleaning, child-rearing, harvesting crops, and respecting husbands and in-laws are the duties of females. If they are not fulfilling their role, they should be punished by their fathers and husbands. (brother-in-law)

Socio-cultural values, beliefs, and customs were identified as contributing factors to violence and suicide among women:

In our culture, it is sadly accepted that women can be abused as a form of punishment, and this act is not seen as a criminal offense. From a young age, we are taught that females are the honor of the family and should be kept under strict supervision. These expectations are also imposed on female in-laws, and if they fail to meet them, they can be penalized by their in-laws without interference from their parents. Tragically, my daughter had no choice in this matter and lost her life as a result. (Father)

### Absence of Women Police station in the area:

The absence of female police stations in the district significantly exacerbates violence against women. This glaring gap in state support emboldens perpetrators, perpetuating a culture of violence and undermining the safety and well-being of women. A brother underscored this issue, stating:

“Since we don’t have a women’s police station in the entire district, families often discourage females from reporting crimes at male-dominated police stations, fearing it will bring shame and stigma upon them.”

### Findings from Observation:

During the research, the observer noticed a woman carrying a bundle of dry stick-on heads and using them to prepare tea and food at home. After eating, she washed dishes and clothes before heading to the field to collect grass. The researcher followed her and sat nearby while she worked. When asked about her feelings towards her life, she expressed that it had become very difficult and overwhelming. She desired freedom but felt restricted by her parents, who did not allow her to seek a divorce. Unfortunately, she saw suicide as the only way to escape her circumstances. (Researcher’s observation)

## DISCUSSION

This research found that domestic violence was the primary cause of suicide among women in Chitral. Additionally, there were various subfactors at the personal, family, community, and societal levels that contributed to domestic violence. Infertility was found to be a personal factor that led to physical and psychological abuse by husbands, as well as threats of divorce by in-laws, in women. This abusive behavior had detrimental effects on the victims, including stress, anxiety, depression, despair, and helplessness, ultimately resulting in suicide. International research indicates that women who fear rejection and divorce are more vulnerable to suicide. Additionally, females who are victims of violence may also have an elevated risk of suicide, as they view it as their only means of escaping the brutality they experience.^2^

Factors such as poor parent bonding, forced marriage, and external relations of the husband played a role in women’s suicides. These factors negatively impacted the family relationships, leading to mental health issues such as worry, hopelessness, and further violence, ultimately resulting in suicide. International research supports these findings, suggesting that children who suffer from poor family support, neglect, and parental loss have significant impacts on suicidal behaviors later in life.^17^

Lack of community support was identified as a significant factor contributing to violence and subsequent suicide among women. Participants revealed that many women face violence from their husbands and in-laws, but they receive no support from their family or the community. The study also found that some victims who tried to speak out against their abusers received no assistance, and were even blamed for their victimization. International research further supports these findings, indicating that women who feel despair and rejection from their community are at a higher risk of self-destructive behavior compared to those who receive social acceptance, support, love, and self-empowerment.^18^,^19^

Cultural norms and customs contribute significantly to violence against women. Society places high expectations on women, requiring them to possess various qualities such as sensibility, selflessness, tolerance, empathy, reliability, and the ability to perform all family-related tasks. Women are often viewed as weak, timid, and lacking decision-making abilities, leading to their assignment of domestic and agricultural responsibilities. When these tasks are left incomplete, women may face violence. Consequently, women are expected to tolerate any situation to avoid the stigma of divorce. This behavior places women at a high risk of experiencing violence and facing the possibility of suicide over time, the burden becomes overwhelming, resulting in fatigue and sickness. Research indicates that in societies with rigid gender roles and a strong association between masculinity and dominance, violence against women is more prevalent.^20^,^21^

Poor policymaking and inadequate implementation by local government contribute to the issue of suicide among women. Women have limited access to reporting their problems and seeking justice, as there are no women’s police stations in the district, and females are not allowed to visit male police officers. As a result, victims of violence may feel compelled to compromise or resort to ending their lives to escape their difficult situations. Another study from Pakistan indicates that despite the government’s commitment, violence against women remains widespread in Pakistan, with many women not reporting or seeking help for the abuse they experience because of their lack of knowledge about their rights and available options for recourse.^22^

### Strength of the Study:

The study’s strengths include its focus on a specific and vulnerable population, its exploration of the complex interplay between various factors contributing to suicide, and its potential to inform evidence-based policy and practice. To build upon this research, future studies could employ a mixed-methods approach, incorporating both quantitative and qualitative data to provide a more comprehensive understanding of the issue. Additionally, researchers could explore the effectiveness of community-based interventions aimed at preventing domestic violence and promoting women’s empowerment.

### Recommendations:

The study’s findings have significant implications for policymakers, law enforcement organizations, and mental health professionals:

- Develop and implement suicide prevention programs tailored to the needs of married women in Chittal.
- Strengthen laws and policies addressing domestic violence and women’s rights.
- Increase access to mental health services and support for women experiencing domestic violence and mental health issues.
- Promote community-based initiatives that foster women’s empowerment and social support networks.

### Limitations

Due to time and financial limitations, the current study was conducted only in rural areas of Pakistan. However, conducting the study with an urban population could provide a better understanding of this phenomenon.

## CONCLUSION

This study focused on the factors contributing to suicide among married women in Chitral, Pakistan. The research revealed that domestic violence was the main cause of suicide among these women. Other factors included lack of empowerment, high expectations, poor family and relationship dynamics, forced or mismatched marriages, and extramarital affairs. These factors created a situation where women felt trapped and unable to escape, leading to feelings of hopelessness and helplessness. The study’s findings can be used by policymakers and law enforcement organizations to develop suicide prevention programs and reduce the high number of suicide cases in Chitral.
